# Ischemia/Reperfusion Injury in Liver Surgery and Transplantation: Pathophysiology

**DOI:** 10.1155/2012/176723

**Published:** 2012-05-30

**Authors:** Kilian Weigand, Sylvia Brost, Niels Steinebrunner, Markus Büchler, Peter Schemmer, Martina Müller

**Affiliations:** ^1^Department of Gastroenterology, Endocrinology, Rheumatology and Infectious Diseases, University Hospital Regensburg, D-93053 Regensburg, Germany; ^2^Department of Gastroenterology, University Hospital Heidelberg, D-69120 Heidelberg, Germany; ^3^Department of General and Transplant Surgery, University Hospital Heidelberg, D-69120 Heidelberg, Germany

## Abstract

Liver ischemia/reperfusion (IR) injury is caused by a heavily toothed network of interactions of cells of the immune system, cytokine production, and reduced microcirculatory blood flow in the liver. These complex networks are further elaborated by multiple intracellular pathways activated by cytokines, chemokines, and danger-associated molecular patterns. Furthermore, intracellular ionic disturbances and especially mitochondrial disorders play an important role leading to apoptosis and necrosis of hepatocytes in IR injury. Overall, enhanced production of reactive oxygen species, found very early in IR injury, plays an important role in liver tissue damage at several points within these complex networks. Many contributors to IR injury are only incompletely understood so far. This paper tempts to give an overview of the different mechanisms involved in the formation of IR injury. Only by further elucidation of these complex mechanisms IR injury can be understood and possible therapeutic strategies can be improved or be developed.

## 1. Introduction

Ischemia/reperfusion (IR) injury of the liver results from a loss of blood supply reducing oxygen supply to the organ. Upon revascularisation the liver undergoes reperfusion injury. Together these factors lead to affection of oxygen-dependent cells within the liver causing impairment of organ function. Affected are all cells requiring mitochondrial oxidative phosphorylation for their metabolism [1]. Warm IR injury can be separated from cold IR injury. Warm IR injury occurs during prolonged surgical liver resection using clamping of the perfusion [2]. Other aetiologies are reduced liver perfusion due to shock, heart failure, respiratory failure, hemorrhage, trauma, and sepsis [3–5]. In contrast, cold IR injury follows liver transplantation with the necessity of cold preservation of the donor organ, followed by reperfusion after implantation [6, 7]. Furthermore, it has been demonstrated that tissue damage occurs in two phases, an early and a late phase [8, 9]. The early phase which occurs within the first 6 hours following reperfusion is thought to be the consequence of the fast change in the redox state of the liver tissue [9, 10]. Most likely, this change is caused by hepatocytes, Kupffer cells (KCs), and sinusoidal endothelial cells (SECs) [8, 9, 11]. In contrast, the late phase of IR injury is caused by the production of cytokines and chemokines followed by the infiltration of leukocytes into the liver tissue [8, 9, 12].

Of clinical relevance is that liver IR injury results in elevated liver enzymes, biliary strictures, clinical dysfunction, or even liver failure [13]. Furthermore, other organs can develop dysfunction secondary to the liver damage. Possible affected organs are lungs, heart, kidneys, and blood vessels [14–17]. Risk factors for IR injury include age of the liver, sex and others [18–21].

A complex network and cross talk of multiple molecular mechanisms and cellular interactions lead to liver IR injury [22, 23]. The result of these processes is cell death by apoptosis and necrosis via different pathways. Redox status, cellular ionic disturbances, cytokines, chemokines, other mediators and molecular mechanisms as well as many different cells like KC, SEC, dendritic cells, leukocytes, and lymphocytes are involved in this process and are closely interlocked. Therefore, there are still many open questions regarding this inflammatory response. This paper tempts to give a systematic overview of the different components and signalling pathways leading to IR injury.

## 2. Altered Redox Status and Reduced Microcirculatory Blood Flow

IR injury starts with reduced blood flow and a lack of oxygen supply [24, 25]. This ischemia leads to a lack of adenosine triphosphate (ATP) production in hepatocytes, KC, and SEC [26]. As a result the function of the ATP-dependent sodium/potassium plasma membrane pump (Na^+^/K^+^ ATPase) is impaired. This results in an increase of intracellular Na^+^, which is followed by a swelling of the hepatocytes, KC and SEC. Narrowing of the sinusoidals is the consequence. Within minutes after reperfusion, enhanced levels of reactive oxygen species (ROS), such as superoxide (O_2_^−^), hydrogen peroxide (H_2_O_2_) and hydroxyl radical (OH^•^), can be measured [8, 9, 11, 27]. Cellular sources for these ROS are mitochondrial metabolism, hepatocyte-derived xanthine oxidase, and KC- and SEC-associated NADPH oxidase [8, 9, 11, 28]. This increase of ROS is accompanied by reduced nitric oxide (NO), most likely due to a decreased function of the NO synthase (NOS) in SEC [29, 30]. Since NO is a vasodilator [31], reduced NO aggravates the sinusoidal narrowing. However, these mechanisms are still controversially discussed [32, 33]. Especially the role of the different NOS isoforms is still unclear [34]. The effects of NO are well known. It increases the sinusoidal diameter and increases intrahepatic ATP levels via better oxygen supply. Thereby mitochondrial damage and leukocyte infiltration are reduced. Since NO is synthesized by NOS, NOS should be protective in IR injury. While this is well observed for the endothelial NOS (eNOS), the role of the inducible NOS (iNOS) is less clear [34]. In the late phase of IR injury it probably is also protective, while in the early phase, there may be a harmful role of iNOS.

Nevertheless, the changes occurring in IR injury lead to a more oxidative environment with the ROS leading to both, apoptotic and necrotic cell death of hepatocytes and SEC [35, 36]. ROS causes damage to membrane lipids resulting in cell swelling and death [35]. The damage is not limited to the plasma membrane but includes cell organelles and extracellular matrix. Besides swelling of cells and reduced NO levels, the described damage leads to an increase of vasoconstrictors like endothelin and thromboxane A2 [37]. In addition, adhesion and aggregation of platelets and leucocytes is increased (see below). This leads to further narrowing of the sinusoidals with significant reduction of microcirculatory blood flow including areas with complete absence of blood flow [38], enhancing the lack of oxygen supply. Increased ROS and decreased NO levels play further roles which will be discussed below.

## 3. Ionic and Mitochondrial Disturbances

In IR injury significant changes of intracellular Ca^2+^ concentration in the hepatocytes can be found [39]. Ca^2+^ is mainly found in three cellular compartments, in the cytosol, the mitochondria, and the endoplasmic reticulum (ER). The homeostatic concentration is regulated by different Ca^2+^ channels. In IR injury cytosolic Ca^2+^ concentration is increased as a result of increased entry across the plasma membrane and release from the ER. Reason for this cytoplasmic Ca^2+^ overload is the activation of the ryanodine receptor in the ER membrane and the so-called transient receptor potential (TRP) channels in the plasma membrane. There is evidence that ROS can activate these channels [40, 41]. Secondary to decreased Ca^2+^ concentration in the endoplasmic reticulum so-called store operated calcium (SOC) channels in the plasma membrane further increase Ca^2+^ influx [42, 43]. In addition, in IR injury the Ca^2+^ ATPase in ER and plasma membrane is inhibited, potentially because of ATP depletion. Normally this Ca^2+^ ATPase discharges cytosolic Ca^2+^ into extracellular space and into the ER counteracting the above mentioned Ca^2+^ channels [44].

Increased cytosolic Ca^2+^ leads to stimulation of the Ca^2+^ uniporter in the mitochondrial membrane [45]. As a result the mitochondrial Ca^2+^ concentration increases as well. The mechanism how the mitochondrial Ca^2+^ uniporter is activated has not been fully resolved, so far. It is believed that the mitochondrial P2Y-like receptor 1 (mP2Y_1_) is activated by adenosine diphosphate (ADP) and adenosine monophosphate (AMP). The mP2Y_1_ stimulates the PLC-dependent mP2Y-like receptor resulting in activation of the Ca^2+^ uniporter [46, 47]. In contrast, mP2Y_2_ activated by ATP leads to inhibition of the uniporter. During IR injury ATP is depleted, as discussed before, possibly leading to activation of the uniporter. As a consequence of this increased mitochondrial Ca^2+^ concentration the mitochondrial transmembrane potential is reduced. To maintain the mitochondrial membrane the mitochondrial ATP-synthase reverses its activity and hydrolyzes ATP to provide energy for different ionic pumps in the mitochondrial membrane [39]. However, this further increases Ca^2+^ influx resulting in ATP consumption instead of production in the mitochondria. This is enhanced by the fact that ROS causes oxidative damage to the enzymes of the respiratory chain in the mitochondria leading to failure of ATP production [48]. Cytosolic and mitochondrial Ca^2+^ and other ionic disturbance lead to damage of plasma and mitochondrial membranes including the formation and opening of mitochondrial permeability transition (MPT) pores [49]. MPT pores are formed from integral not fully identified mitochondrial membrane proteins [50, 51]. Hepatic mitochondria afflicted by MPT pores are permanently damaged due to depolarization of the mitochondria [37]. When only a few mitochondria are afflicted, they are removed from the hepatocyte by lysosomal mitophagy [52]. Such damaged mitochondria are a source for further ROS production and ATP consumption [53]. However, ROS themselves induce MPT pore opening. With the number of damaged mitochondria increasing, cytochrome C is released from the mitochondria into the cytosol triggering apoptosis [48]. When the majority of the mitochondria within the hepatocyte are afflicted by MPT pores ATP levels drop too fast resulting in cell death by necrosis [49, 54].

Other important ionic disturbances in IR injury include intracellular Na^+^ and hydrogen (H^+^) concentrations. Lack of oxygen supply leads to anaerobic respiration of the hepatocytes resulting in intracellular acidosis [55]. To stabilize intracellular pH within normal range the Na^+^/H^+^ exchanger is activated by the hepatocytes, resulting in reduced cytosolic H^+^ and further increased Na^+^ levels. In addition, the Na^+^/K^+^ exchanger is ATP dependent, so ATP depletion, as in IR injury, subsequently blocks this exchange leading to further increase of intracellular Na^+^ concentration resulting in cell death [56].

**Figure 1:**
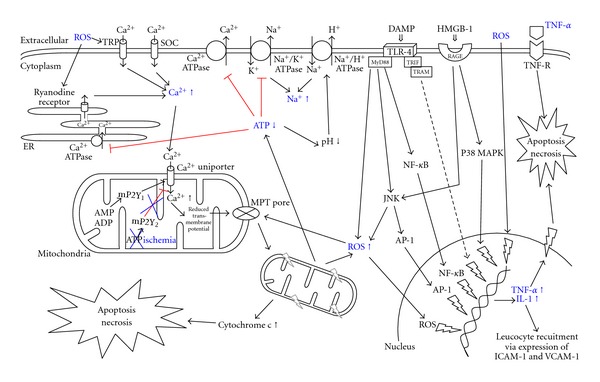
Intracellular signalling pathways and ionic disturbances engaged during IR injury, resulting in cellular swelling, apoptosis, and necrosis. ADP: adenosine diphosphate; AMP: adenosine monophosphate; AP-1: activator protein-1; ATP: adenosine triphosphate; DAMP: danger-associated molecular pattern; HMGB-1: high mobility group box-1; ICAM-1: intercellular adhesion molecule-1; IL-1: interleukin-1; JNK: c-Jun N-terminal kinase; MAPK: mitogen-activated protein kinase; MPT pore: mitochondrial permeability transition pore; MyD88: myeloid differentiation factor 88; NF-*κ*B: nuclear factor kappa B; RAGE: receptor for advanced gylcation end product; ROS: reactive oxygen species; SOC: store operated calcium channel; TLR: toll-like receptor; TNF: tumor necrosis factor; TRAM: TRIF-related adaptor molecule; TRIF: TIR domain-containing adaptor inducing interferon; TRP: transient receptor protein; VCAM-1: vascular adhesion molecule-1.

Furthermore, this counteracts the protective effect of an acidic pH during reperfusion [57], for example, the maintenance of an acidic pH prevents the formation of MPT pores [49]. However, these regulations are still based on experimental observations and need to be studied further to understand the relevance in IR injury (Figure 1).

## 4. Cellular Cascade in IR Injury

Many different cell types are involved in the process of hepatic damage and cell death in IR injury. The key cells initiating IR injury are the KCs [58–60]. Besides their direct damage by ROS, as discussed above, they are also activated by ROS leading to production of more ROS and thereby entering a cycle of self-activation and -destruction. In addition, KC are activated by the systemic complement system [61] which may also be liberated by damaged hepatocytes. In addition, complement leads to further liver damage by formation of a membrane attack complex in the plasma membrane, lysing liver cells [62].

Activated KCs also produce proinflammatory cytokines including interleukin-1*β* (IL-1*β*) and tumor necrosis factor-*α* (TNF-*α*) [63]. These cytokines lead to activation and migration of neutrophils and CD4+ T lymphocytes into the liver [64]. Furthermore, these cytokines stimulate SEC and hepatocytes to produce ROS and to express adhesion molecules on the cell surface [65]. As described above, this leads to adhesion and aggregation of leucocytes and platelets [66], influencing the microcirculatory blood flow in the liver.

The recruitment of neutrophils and CD4+ T lymphocytes is further enhanced by the matrix metalloproteinase 9 [67] after ischemic damage of the liver. Via production of interferon-*γ* (IFN-*γ*) and IL-17 by activated CD4+ T lymphocytes additional activation of KC and hepatocytes is achieved [68, 69]. Thus CD4+ T lymphocytes and KC reciprocally activate each other [64]. These chemokines furthermore activate natural killer T (NKT) cells. Activated NKT cells directly damage liver tissue and also produce IFN-*γ* with further activation of KC and hepatocytes [69, 70]. The net result of this circular activation and stimulation of different cell sub types is destruction of hepatocytes and SEC [71, 72].

**Figure 2:**
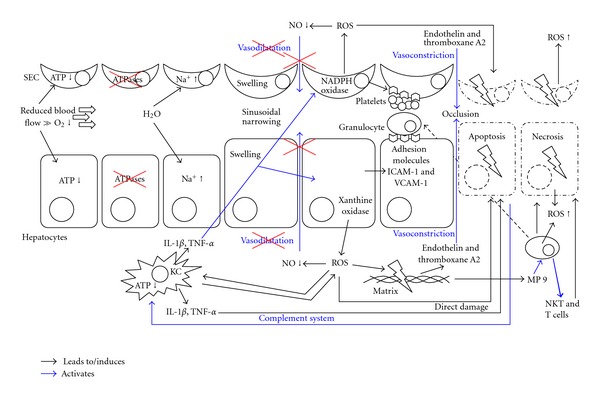
Cellular interaction involved in IR injury, resulting in cellular swelling, apoptosis, and necrosis. ATP: adenosine triphosphate; ICAM-1: intercellular adhesion molecule-1; KC: Kupffer cell; IL-1, interleukin-1; NKT: natural killer T cell; NO: nitric oxide; ROS: reactive oxygen species; SEC: sinusoidal endothelial cells; T cell: CD4+ T lymphocyte; TNF: tumor necrosis factor; VCAM-1: vascular adhesion molecule-1.

The expressed cell-surface adhesion molecules on hepatocytes and SEC include intercellular adhesion molecule-1 (ICAM-1) and vascular adhesion molecule-1 (VCAM-1) [28, 73]. Neutrophils bind to ICAM-1 and VCAM-1 and by doing so migrate across the endothelium into the liver parenchyma enhancing ROS production and degranulation of cytoplasmic vesicles containing enzymes capable to degrade extracellular matrix and hepatocytes [28] (Figure 2).

## 5. Death Signalling Pathways

Besides direct damage of hepatocytes by neutrophils, NKT cells, the complement system and ROS, the main destruction of cells is mediated by endogeneous pathways leading to apoptosis or necrosis of hepatic cells during IR injury.

This paper is not capable to focus on all cytokine cascades with pro- and antiinflammatory effects [74] as well as their effect during IR injury, but will concentrate on some important signalling pathways. The most important component in IR injury seems to be TNF-*α* [75, 76]. The pathways leading to up regulation of TNF-*α* have been described above. TNF-*α* binds to specific TNF-receptors, as for example TNF-R1 and TNF-R2, on the hepatocyte surface which leads to increased production of cytokines and ROS. In addition, activation of CD95 leads to apoptosis [77–79]. Furthermore, CD95 also binds NKT cells leading to direct destruction of hepatocytes [80].

Furthermore, downstream of the receptor the nuclear factor kappa B (NF-*κ*B), the mitogen-activated protein kinase (MAPK) and c-Jun N-terminal kinase (JNK) are activated [81–83]. The various cytokines and the mentioned molecules lead to alteration of various factors further downstream like transcription factors, activator protein-1 (AP-1), heat shock factor, signal transducer and activators of transcription (STATs), antioxidants, inflammation-stimulated inducible enzymes (COX-2), intracellular signalling molecules, antiapoptotic proteins (Bcl-2, Bcl-x), and many more [37]. The damage in IR injury therefore spreads throughout the entire cell. NF-*κ*B furthermore upregulates the expression of cytokines, like TNF-*α* [84], and of ICAM-1 and VCAM-1 [75, 82], enhancing the recruitment of neutrophils. AP-1 promotes apoptosis of liver cells by activation of caspase-3 and release of cytochrome C [82].

ROS furthermore inherit a direct oxidative damage of DNA within the nucleus resulting in further failure of protein transcription and translation. In addition, ROS cause posttranslational protein modification [85]. These alterations and pathways lead to apoptosis of the damaged cells.

The intracellular damage and alterations as well as the damage of extracellular matrix are followed by the release of danger-associated molecular patterns (DAMPs). Examples of DAMPs released during IR injury are the nuclear transcription factor high mobility group box-1 (HMGB-1), the cytoplasmic Ca^2+^ regulator S100, ATP, DNA, and hyaluronic acid [86–90]. DAMPs bind to a group of so-called pattern recognition receptors (PRRs) on the cell surface as well as in the cytoplasm [37]. In IR injury mainly two PRRs are involved, the toll-like receptors (TLRs), specifically TLR-4, and the receptor for advanced glycation end products (RAGE). To present knowledge TLR-4 provides an important link between liver damage and activation of the immune system. Activation of TLR-4 triggers intracellular signalling cascades in IR injury [91]. The Toll-IL-1 receptor domain (TIR) of TLR-4 interacts with intracellular adaptors. One may be the myeloid differentiation factor 88 (MyD88), others are TIR domain-containing adaptor inducing interferon-*β* (TRIF), and TRIF-related adaptor molecule (TRAM) [92]. Via production of proinflammatory cytokines the inflammatory response is mediated leading to liver IR injury [91–93]. Included in the downstream process of these activation are further transcription factors like NF-*κ*B, AP-1, STAT, the MAP kinase JNK, and ROS [86, 94–96].

The best characterized DAMP is HMGB-1 which is expressed by all nucleated cells within the liver and is released upon necrosis and apoptosis [86, 97]. HMGB-1 binding to RAGE in IR injury leads to a signalling cascade involving activation of JNK and other kinases, increasing expression and activation of the inducible transcription factor early growth response-1. As a consequence the upregulation of several gene families is found, recruiting immune cells into the post ischemic liver [98]. RAGE is mainly expressed on dendritic cells and to lesser extent on KC [98]. Furthermore, dendritic cells and KC also express TLR-4 [99]. This hints to an important, but at present unclear, function of dendritic cells during IR injury of the liver.

This complex communication of the described networks is responsible to initiate and propagate IR injury.

## 6. Conclusions

The understanding of the molecular mechanisms underlying cell death in hepatic IR injury will provide the basis for the development of new strategies for inhibition of liver injury and improvement of survival of the graft. The initial phase of IR injury involves the release of ROS and proinflammatory mediators by KC. ROS lead to oxidative damage, induction of p53, apoptosis and necrosis of hepatocytes and endothelial cells. The late phase (6–48 hours after reperfusion) is characterized by neutrophil-mediated inflammatory responses. Thus, proteins regulating the cellular redox equilibrium, p53-dependent apoptosis and cellular death receptors represent potential targets for novel pharmaceutical interventions to protect hepatocytes from IR injury-induced cell death.
